# Pubic Symphysis Stress Reaction and Pelvic Girdle Myositis Following Robotic-Assisted Inguinal Hernia Repair: A Case Report and Brief Review of the Literature

**DOI:** 10.7759/cureus.112352

**Published:** 2026-07-09

**Authors:** Francisco M Torres, Emory Hubbard

**Affiliations:** 1 Physical Medicine and Rehabilitation, Morton Plant Hospital, Clearwater, USA; 2 Applied Physiology and Kinesiology, University of Florida, Gainesville, USA

**Keywords:** chronic postoperative pain, groin pain, inguinal-related groin pain, magnetic resonance (mr), mri, musculoskeletal rehabilitation, myositis, physical therapy compliance, pubic symphysis edema, robotic-assisted inguinal hernia repair

## Abstract

This report presents an unusual case of persistent groin and lower abdominal pain following robotic-assisted inguinal hernia repair (R-IHR), along with a brief review of the literature. To our knowledge, this is the first reported case of pubic symphysis stress reaction with adjacent pelvic girdle myositis following R-IHR, a musculoskeletal complication pairing not previously described in the hernia repair or robotic surgery literature. Imaging findings revealed edema and capsular hypertrophy at the pubic symphysis with diffuse pelvic girdle myositis on magnetic resonance imaging (MRI), findings not captured on computed tomography (CT), suggesting a postoperative biomechanical mechanism.

The specific robotic surgical technique (transabdominal preperitoneal vs. totally extraperitoneal) used in this case could not be confirmed, as the original operative report was not available to the authors at the time of this report; the general term R-IHR is used throughout to reflect this uncertainty.

A 41-year-old physically active man presented more than one year after R-IHR, complaining of progressively worsening right groin and lower abdominal pain. The pain was moderate to severe, dull, and aching, with intermittent sharp, stabbing episodes. It was exacerbated by bending, coughing, bowel movements, prolonged sitting, recumbency, exercise, and sexual activity. He reported no weakness, bowel or bladder dysfunction, or systemic symptoms. Examination revealed focal tenderness in the right inguinal region and right lower abdominal quadrant without rebound tenderness, no signs of psoas involvement, and normal strength, range of motion, neurologic function, and gait. Pelvic CT was unremarkable, showing only bilateral hydroceles. Pelvic MRI demonstrated edema and capsular hypertrophy along both sides of the pubic symphysis, with multifocal intramuscular enhancement in the right gluteus medius, bilateral adductor muscles, and gluteus maximus, consistent with myositis. Inflammatory laboratory workup was negative. The Douleur Neuropathique 4 questionnaire indicated nociceptive rather than neuropathic pain. These findings suggest that biomechanical stress and microtrauma to the pubic symphysis during surgery initiated a local inflammatory cascade. The patient had declined postoperative physical therapy, citing high baseline fitness; prolonged activity restriction over 12 months provided no relief.

Persistent groin pain following R-IHR warrants consideration of pubic symphysis stress reaction and adjacent myositis, particularly when CT is nondiagnostic and neurologic examination is normal. MRI may be necessary to identify underlying musculoskeletal pathology and guide management. This case further highlights the clinical risk of physically fit patients declining structured rehabilitation; general conditioning does not substitute for targeted postoperative physical therapy.

## Introduction

Inguinal hernia repair is among the most commonly performed operations worldwide, with an estimated 20 million procedures performed annually [1]. The adoption of minimally invasive techniques, including laparoscopic and, more recently, robotic-assisted approaches, has been driven in part by evidence suggesting reduced acute postoperative pain compared with open repair [2,3]. Nevertheless, chronic postoperative inguinal pain (CPIP), defined by the HerniaSurge Group as pain of at least moderate severity affecting daily activities beyond three months postoperatively, remains a persistent problem, with reported incidence ranging from 6% to more than 60% across studies depending on surgical technique, follow-up duration, and pain definition employed [4-6].

Robotic-assisted inguinal hernia repair (R-IHR) is increasingly utilized despite limited evidence of superior clinical outcomes compared with standard laparoscopic repair. A 2024 meta-analysis comparing robot-assisted and laparoscopic inguinal hernia repair across 15 studies and more than 64,000 patients found no statistically significant differences in overall complication rates or surgical site infection rates, and noted significantly longer operative times with the robotic approach [7]. Similarly, a systematic review and meta-analysis encompassing data through July 2022 reported that R-IHR was associated with lower hernia recurrence but with a postoperative complication profile comparable with that of laparoscopic repair [8].

CPIP is most often attributed to neuropathic mechanisms involving the ilioinguinal, iliohypogastric, or genitofemoral nerves, or to mechanical complications related to mesh fixation [4,9]. Musculoskeletal sources, including stress reactions of the pubic symphysis, osteitis pubis, and myositis of the pelvic girdle musculature, are described predominantly in the context of pelvic or urological surgery and in athletes, but have not been well characterized as a sequela of inguinal hernia repair [10,11]. When computed tomography (CT) imaging is nondiagnostic and neurological examination is normal, magnetic resonance imaging (MRI) of the pelvis may reveal underlying musculoskeletal pathology that substantially changes the diagnostic and therapeutic approach.

The pubic symphysis is a nonsynovial amphiarthrodial joint serving as the anterior fulcrum of the pelvic ring, subject to competing tensile forces from the rectus abdominis superiorly and the adductor complex inferiorly. Pubic symphysis stress reaction arises when repetitive microtrauma or acute biomechanical disruption exceeds the capacity for osseous repair, producing a spectrum of pathology ranging from periarticular edema and capsular hypertrophy to subchondral bone marrow edema, articular surface irregularity, and, in advanced cases, frank stress fracture [12]. Clinically, patients present with focal anterior pelvic and groin pain exacerbated by weight-bearing, resisted hip adduction, and activities that load the anterior abdominal wall. Plain radiographs are frequently normal in early disease; MRI is the modality of choice, demonstrating subchondral edema, periarticular soft-tissue signal changes, and associated adductor or rectus abdominis enthesopathy [12,13]. CT provides complementary detail in chronic cases where cortical irregularity, subchondral sclerosis, or osteophyte formation is suspected but not clearly delineated on MRI [13]. Pelvic girdle myositis, as observed in the present case, represents inflammatory involvement of the surrounding musculature, the adductors, gluteus medius, and gluteus maximus, likely secondary to the same biomechanical insult that stressed the symphysis, resulting in persistent regional pain, trigger point formation, restricted hip range of motion, and, in some cases, symptoms referrable to the perineum or inguinal region [14]. On MRI, myositis is identified by intramuscular T2 signal hyperintensity and postcontrast enhancement in the affected muscle groups, findings that are not reliably captured on CT [13,14].

To the best of our knowledge, this presentation has not been previously reported in the context of inguinal hernia repair. We conducted a structured literature search using PubMed, MEDLINE, and Embase with the following search terms in combination: "pubic symphysis", "myositis", "osteitis pubis", "inguinal hernia repair", "robotic hernia repair", "chronic postoperative inguinal pain", and "pelvic girdle". No previously published reports were identified describing pubic symphysis stress reaction or adjacent pelvic girdle myositis as a complication of inguinal hernia repair, robotic-assisted or otherwise. The available literature associates pubic symphysis pathology primarily with athletic pubalgia, urological and gynecological procedures, and parturition - contexts in which biomechanical disruption of the anterior pelvic ring is well established [10-15]. While biomechanical alterations in abdominal wall dynamics following hernia repair have been proposed as a mechanism for groin pain, a direct relationship between hernia repair and pubic symphysis stress reaction with adjacent myositis has not previously been documented.

We present a case of prolonged moderate-to-severe groin and lower abdominal pain following R-IHR in which pelvic MRI demonstrated pubic symphysis edema with capsular hypertrophy and multifocal pelvic girdle myositis, findings not captured on CT. We further review the literature on the clinical and behavioral patterns of physically active patients who decline structured postoperative rehabilitation because of confidence in their baseline fitness, and discuss evidence that general physical conditioning does not substitute for targeted, pathology-specific physical therapy.

## Case presentation

A 41-year-old physically active man presented to a specialized pain management practice with a chief complaint of progressive right groin and lower abdominal pain that had been present since shortly after R-IHR performed more than one year prior. The patient described the pain as moderate to severe, predominantly dull, and aching in character, with superimposed intermittent sharp and stabbing episodes. Pain was consistently exacerbated by bending, coughing, bowel movements, prolonged sitting, recumbency, exercise, and sexual activity. He denied weakness, bowel or bladder dysfunction, or any systemic symptoms.

Physical examination demonstrated focal tenderness in the right inguinal region and right lower abdominal quadrant without rebound tenderness. Gait assessment, pelvic alignment, passive and active range of motion of the bilateral hips and lumbar spine, and muscle tenderness grading of the adductor and gluteal compartments were all within normal limits. There were no clinical signs of psoas involvement. Neurological examination was normal, with preserved strength, sensation, and reflexes bilaterally.

**Figure 1:**
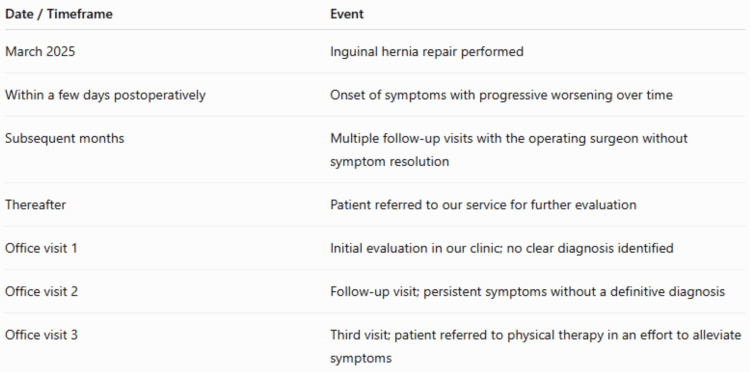
Chronological timeline table

Standardized outcome measures were administered at each of the three consultation visits. The patient consistently reported a pain intensity of 8 out of 10 on the Numeric Rating Scale, indicating severe pain that remained unchanged across visits despite ongoing activity restriction. Functional limitation was assessed using the Patient-Specific Functional Scale, a validated, patient-reported outcome measure with established reliability and responsiveness across musculoskeletal conditions [16]. The patient identified significant functional impairment across multiple activities of daily living, including sleeping, dressing, household tasks, yard work, sports participation, and recreational activities, all of which were scored as substantially limited relative to his presurgical baseline. No standardized quality-of-life or physical performance measures were administered. Figure 1 shows a chronological timeline table.

The patient reported that the pain began within days of the R-IHR procedure. Following surgery, his treating surgeon attributed the pain to muscle overuse and recommended activity restriction. The patient declined a referral for physical therapy, citing his high baseline level of physical fitness as grounds for expecting spontaneous recovery. When formal physiotherapy was declined, no structured home exercise program or written activity modification protocol was prescribed as an alternative, as the absence of a confirmed diagnosis at initial presentation limited the specificity of guidance that could be offered. General activity modification, already in place for over one year per the operating surgeon's recommendation, had yielded no improvement. This underscores a broader clinical challenge: in patients who refuse targeted rehabilitation, the temptation to default to rest must be resisted, as rest alone does not address the underlying neuromuscular and inflammatory pathology.

Pelvic CT was performed and was unremarkable, demonstrating only bilateral hydroceles without evidence of recurrent hernia, mesh complication, hematoma, or bony pathology. Given the persistence of symptoms and normal CT findings, pelvic MRI with contrast was obtained.

**Figure 2:**
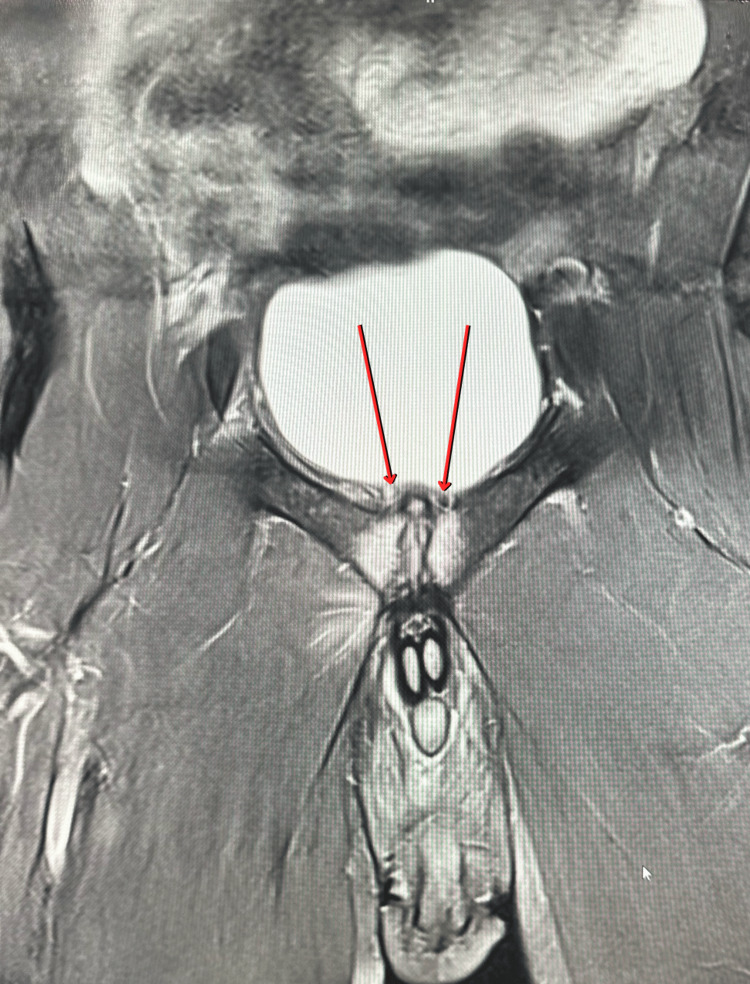
Coronal pelvic MRI (T2 fat-saturated/STIR sequences), demonstrating pubic symphysis edema (see red arrows) MRI: magnetic resonance imaging; STIR: short-tau inversion recovery

**Figure 3:**
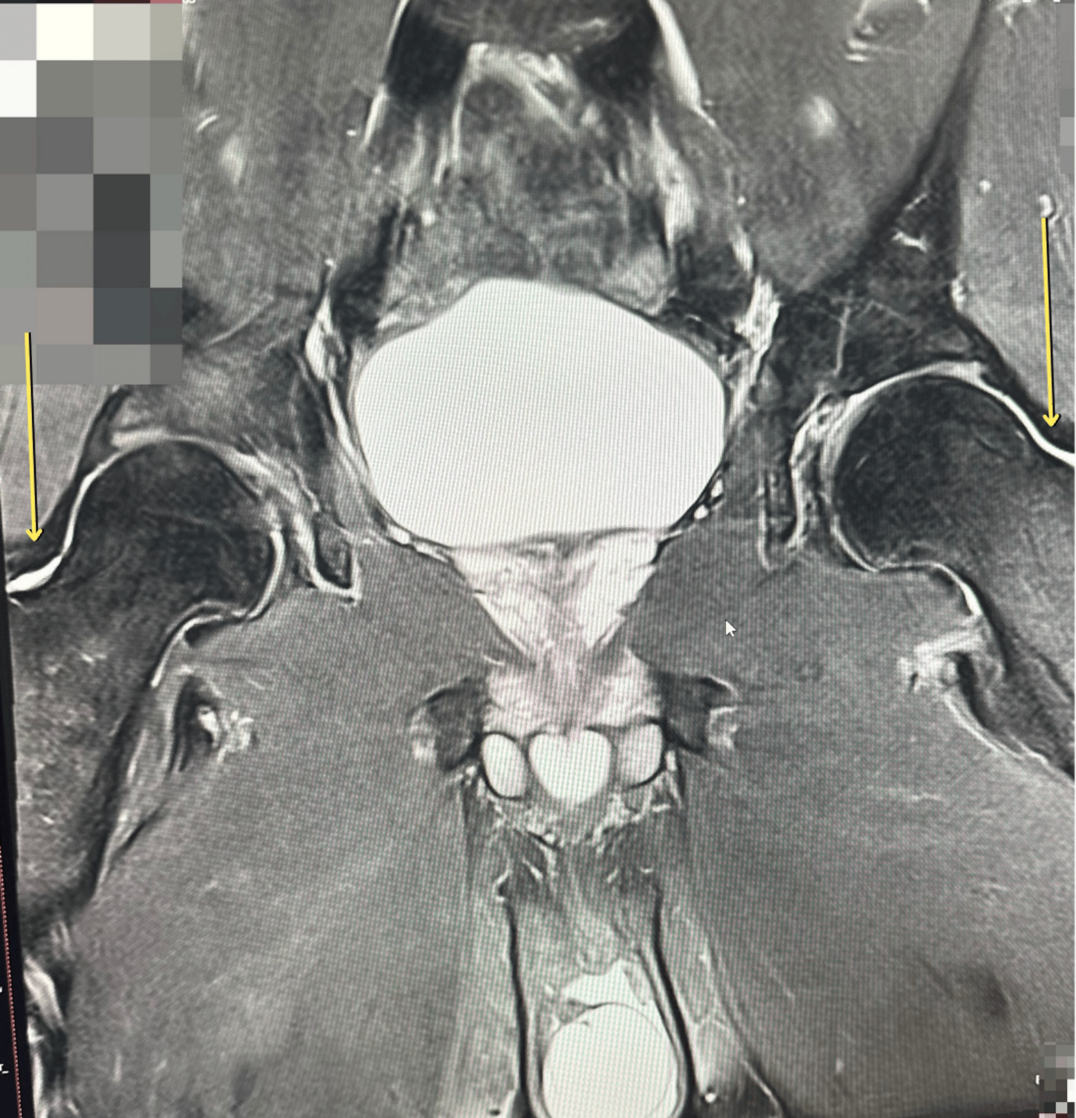
Coronal pelvic MRI (T2 fat-saturated/STIR sequences), demonstrating capsular hypertrophy (see yellow arrows) MRI: magnetic resonance imaging; STIR: short-tau inversion recovery

**Figure 4:**
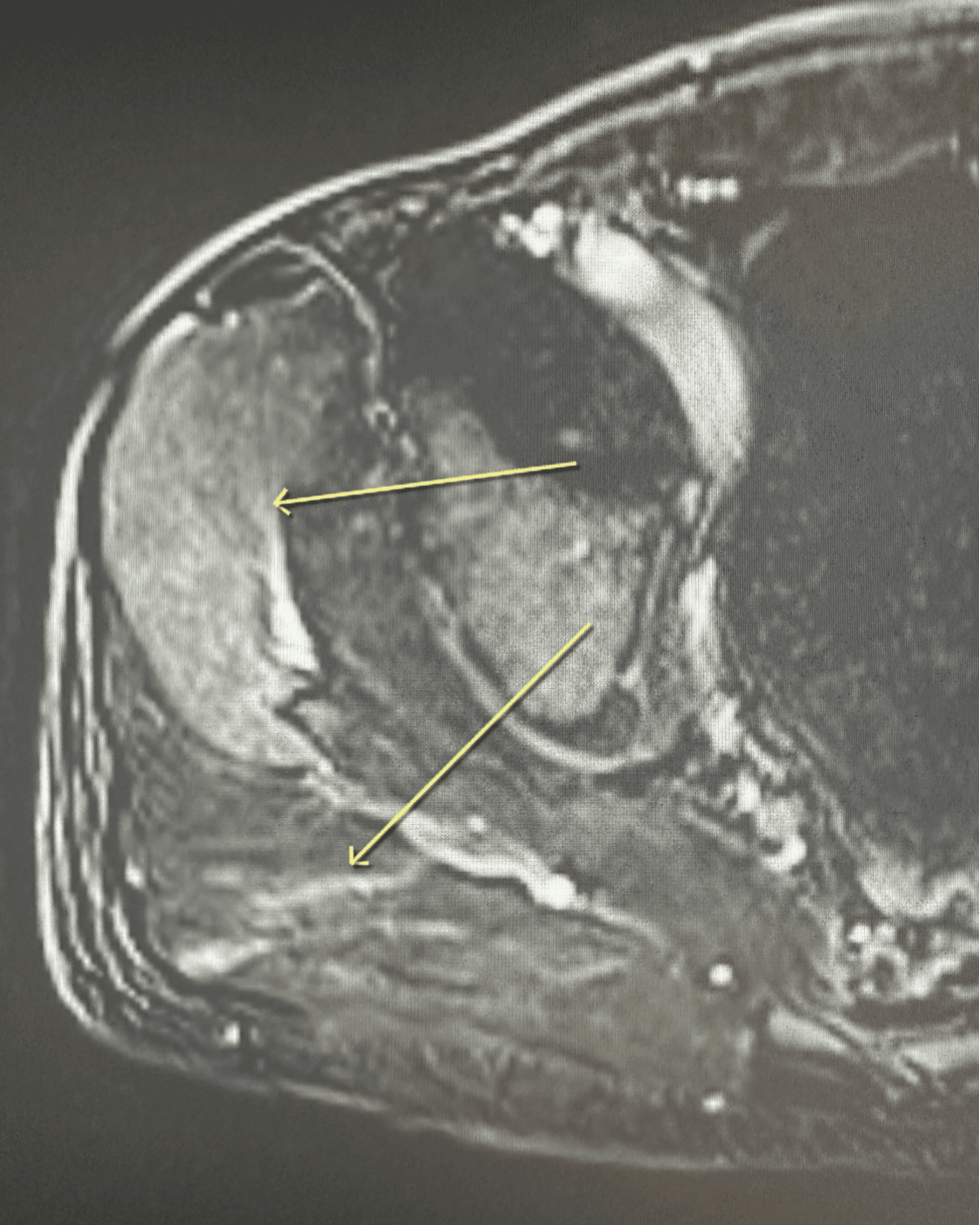
Axial pelvic MRI (postcontrast T1 fat-saturated), demonstrating multifocal intramuscular enhancement in gluteus maximus and minimus (see yellow arrows) MRI: magnetic resonance imaging

**Figure 5:**
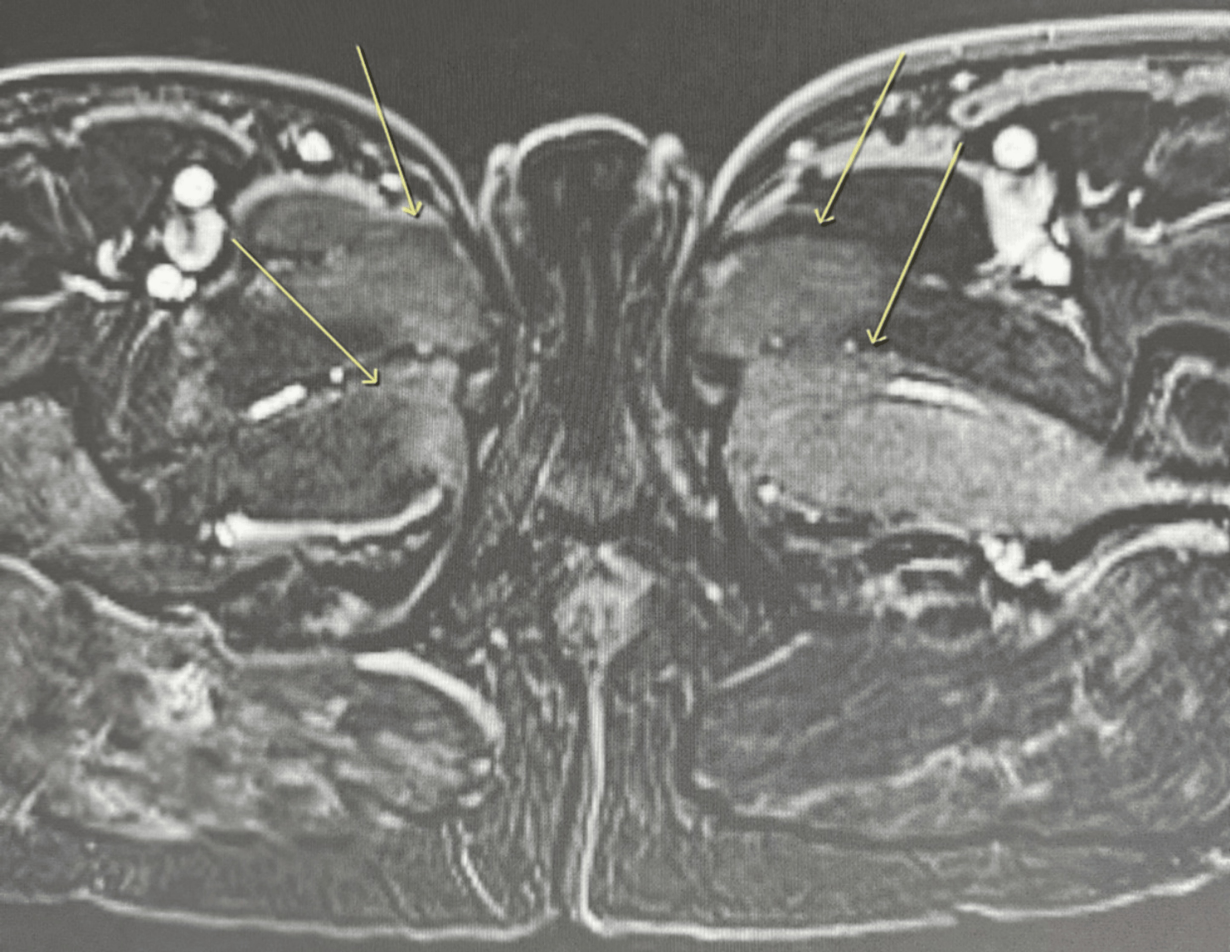
Axial pelvic MRI (postcontrast T1 fat-saturated), demonstrating multifocal intramuscular enhancement in bilateral adductor muscles (see yellow arrows) MRI: magnetic resonance imaging

MRI demonstrated edema and capsular hypertrophy along both sides of the pubic symphysis, as well as multifocal intramuscular enhancement involving the right gluteus medius, bilateral adductor muscles, and gluteus maximus, collectively consistent with myositis (Figures 2-5).

Inflammatory laboratory workup, including complete blood count, C-reactive protein, and erythrocyte sedimentation rate, was within normal limits, effectively excluding systemic inflammatory or infectious etiology. Administration of the Douleur Neuropathique 4 (DN4) questionnaire yielded results strongly indicative of nociceptive rather than neuropathic pain [15].

The constellation of imaging findings, pubic symphysis edema, capsular hypertrophy, and adjacent multifocal myositis (Figures 2-5), in the absence of systemic inflammation, normal neurological examination, and nondiagnostic CT, suggests that biomechanical stress and microtrauma applied to the pubic symphysis and surrounding pelvic girdle musculature during operative dissection or trocar placement may have initiated a local inflammatory cascade. The patient's prolonged period of enforced inactivity, without targeted rehabilitation, likely compounded functional decline without addressing the underlying musculoskeletal pathology.

Prior literature supports the rationale for targeted rehabilitation in pubic symphysis-related pathology. Studies of athletic groin pain demonstrate that structured physiotherapy emphasizing lumbopelvic stabilization, hip girdle strengthening, and adductor conditioning improves pain, function, and return-to-activity rates. However, these approaches have not been specifically validated in patients with pubic symphysis dysfunction following hernia repair, and prospective studies are needed to confirm their efficacy in this population. The absence of functional outcome data in the present case represents a limitation, and no causal relationship between activity restriction and functional decline can be established from this report alone.

No rehabilitation intervention had been administered at the time of this report. The patient declined a physical therapy referral on two occasions before agreeing to an evaluation at a third visit; outcomes are pending. The recommendations regarding physiotherapy are therefore based on clinical rationale, the emerging literature on CPIP management, and the documented consequences of rehabilitation nonadherence, not on observed outcomes in this patient. Prospective studies are needed to establish the efficacy of targeted pelvic floor physiotherapy for this specific complication. Our clinical role in this case was that of a consulting service; no interval follow-up data are available, as treatment is ongoing.

## Discussion

CPIP: mechanisms and epidemiology

CPIP is one of the most clinically significant long-term complications of inguinal hernia repair. An estimated 10%-15% of patients will experience persistent postoperative pain lasting months to years [1]. In a systematic review published in 2022, the incidence of CPIP varied markedly, from 6% to 64.3%, depending on follow-up duration, surgical approach, and the pain definition applied [5]. Risk factors identified by meta-analysis include younger age, female sex, preoperative groin pain, recurrent hernia, and significant early postoperative pain [4].

Neuropathic mechanisms, including injury to the ilioinguinal, iliohypogastric, or genitofemoral nerves, are conventionally considered the primary drivers of CPIP, particularly after open repair. Laparoscopic and robotic approaches generally carry lower rates of CPIP than open mesh repair techniques such as Lichtenstein repair, in part because of reduced nerve exposure and altered fixation methods [4,5]. Robotic-assisted repair remains an area of active investigation; however, technique-specific complications, including those of musculoskeletal origin, are incompletely characterized in the existing literature [5].

Musculoskeletal sources of chronic groin pain after pelvic surgery include osteitis pubis, pubic symphysis stress reaction, and myositis of the adductor and gluteal muscle groups. These entities are well described in athletes and following urological and gynecological procedures, but are rarely reported as complications of inguinal hernia repair [10,11,17]. Acute osteomyelitis of the pubic symphysis following open inguinal hernia repair has been described as a rare infectious complication [17], and MRI-demonstrated pubic symphysis edema with adductor myositis has been reported after robotic-assisted radical prostatectomy [10]. The present case appears to represent a noninfectious, biomechanically mediated variant of pubic symphysis stress reaction with adjacent myositis in the context of R-IHR, a presentation not previously documented in the literature to the authors' knowledge.

Diagnostic considerations

The negative CT in this case, combined with a normal neurological examination and noninflammatory bloodwork, illustrates a diagnostic gap that is not uncommon in CPIP. Standard imaging protocols following hernia repair typically do not include MRI. Yet, this case demonstrates that CT can fail to capture the extent of soft-tissue and osseous inflammation visible on MRI, particularly in early or noninfectious pathology of the pubic symphysis and adjacent musculature (Figures 2, 3).

The DN4 questionnaire findings are clinically noteworthy. A nociceptive profile distinguishes this presentation from the more commonly discussed neuropathic CPIP and has direct management implications, as neuropathic agents (gabapentinoids, tricyclics) would be less appropriate first-line therapy. Targeted physical therapy addressing pelvic girdle biomechanics, activity modification, and potentially image-guided corticosteroid or regenerative injection to the pubic symphysis represents more anatomically directed treatment options for this phenotype.

Based on the pathoanatomy of pubic symphysis stress reaction and the established rehabilitation literature for analogous conditions, a structured, phased physiotherapy approach is clinically appropriate for this presentation. The acute phase prioritizes pain control and load reduction: relative rest from aggravating activities (bending, loaded hip abduction, and high-impact exercise). Manual therapy targeting myofascial trigger points in the adductor and gluteal compartments, and restoration of hip and thoracic range of motion, are introduced as pain allows [18-20]. A case series by McAleer et al. documented that in professional soccer players with MRI-confirmed pubic bone stress, a rehabilitation program targeting adductor strengthening, trunk and lumbopelvic stability, and hip range of motion resulted in resolved or substantially reduced pain and return to full activity in all five subjects, with return-to-training averaging 40.6 days [18]. Verrall et al. described a staged protocol for elite athletes with osteitis pubis structured around pain-free functional milestones, from rest through pain-free ambulation, jogging, sprinting, and multidirectional loading, with return to competition averaging 86 days [19]. In the nonathletic postoperative context, the goals are appropriately reframed: restoration of pain-free activities of daily living, progressive reloading of the pelvic girdle, and correction of compensatory movement patterns that may have developed during prolonged activity restriction. Where formal physiotherapy is unavailable or initially declined, minimum interim guidance should include pelvic girdle mobility exercises, isometric adductor holds within a pain-free range, and avoidance of the specific aggravating postures documented for this patient (prolonged sitting and loaded hip flexion). In this patient, the combination of delayed diagnosis, surgeon-recommended prolonged inactivity, and patient refusal of physiotherapy created compounding barriers to recovery that structured early rehabilitation might have mitigated.

The role of physiotherapy in CPIP

A central question raised by this case is whether structured physiotherapy could have improved the outcome in the observed biomechanical injury pattern. Physiotherapy has historically been understudied in patients undergoing inguinal hernia repair, with most CPIP management research focused on pharmacological and surgical interventions. Early data from multidisciplinary approaches are encouraging: a 2025 prospective study of a one-stop multidisciplinary CPIP clinic, incorporating an abdominal wall surgeon, pain management consultants, and pain specialist physiotherapists, demonstrated marked improvement in both pain scores (Visual Analog Scale 7.2-2.8) and functional activity measures (Modified Activities Assessment Scale 20.3-9.7; p < 0.0001) among patients who received individualized management plans, which commonly included physiotherapy combined with pharmacological or procedural care [21].

With respect to perioperative physiotherapy specifically, a 2025 pilot randomized controlled trial examining perioperative rehabilitation for inguinal hernia repair found the program feasible in terms of recruitment, assessment, and protocol implementation. Patients randomized to the intervention, six weeks of exercise and education before and after surgery, reported less pain on average than control patients at 12 weeks postoperatively, supporting the hypothesis that structured perioperative rehabilitation may reduce the risk and severity of persistent postoperative pain [1].

The patient's baseline level of physical fitness, while high, was characterized by general conditioning rather than targeted pelvic floor and core stabilization. This is clinically important because high levels of general activity and muscle strength do not prevent maladaptive movement patterns, as compensatory motor recruitment can persist after injury or surgery, even in highly trained individuals. Physiotherapy is not only about strengthening muscles but is fundamentally about retraining them. Physiotherapy protocols focused on hip girdle strength, adductor conditioning, and pelvic floor motor control may theoretically yield better outcomes for pubic symphysis-related pathology following hernia repair, though this hypothesis requires prospective investigation.

The patient's refusal of postoperative physical therapy, based on the reasonable but incorrect assumption that general fitness would confer protection, illustrates a clinical education gap that clinicians should proactively anticipate. Physically active patients are a distinct behavioral subset: rather than declining rehabilitation out of fear of movement or logistical barriers, they may do so out of overconfidence in their conditioning. Explicit counseling at the time of surgical planning that postoperative physiotherapy is a pathology-specific, neuromuscular retraining intervention, not a measure of fitness, is an important component of informed perioperative care for this population.

While the imaging findings are consistent with a biomechanically mediated injury pattern, the precise mechanism cannot be confirmed, and alternative explanations, including a preexisting subclinical vulnerability of the pubic symphysis exacerbated by the perioperative period, cannot be excluded.

## Conclusions

This case documents an unusual and previously unreported presentation of pubic symphysis stress reaction with multifocal pelvic girdle myositis as a cause of prolonged moderate-to-severe groin and lower abdominal pain following R-IHR. The findings were identified exclusively on MRI; CT was nondiagnostic, and the neurological examination was normal. Seronegative inflammatory markers and a nociceptive pain profile on the DN4 questionnaire together argue against neuropathic or systemic inflammatory mechanisms and support a biomechanical etiology.

This case reinforces three clinical points: 1) MRI should be considered when CT is nondiagnostic in the setting of persistent postherniorrhaphy groin pain with a normal neurological examination; 2) pain phenotyping using validated tools such as the DN4 questionnaire has direct therapeutic implications, distinguishing nociceptive from neuropathic mechanisms and guiding management accordingly; and 3) this case suggests that early physiotherapy referral, particularly in physically active patients who may underestimate its value, warrants consideration in postoperative planning after inguinal hernia repair, and that prospective studies are needed to evaluate its impact on musculoskeletal outcomes in this population.
